# On the Fringe of the Empire

**Published:** 1918-04-13

**Authors:** 


### On the Fringe of the Empire.

A correspondent sends us the following account of a corner of the Medical Service on the North-West Frontier of India: "We drove the other day to return the S.'s call. He and she are both doctors at the C.M.S. hospital near the [Peshawar] city gates. Mrs. S. took us over the hospital, which adjoins their compound. They have 100 beds for ordinary native patients from the city, etc., but besides this they have a quadrangle surrounded by about twenty little rooms, where patients who are too wild and frightened to go into the ordinary wards are allowed to come *with* their family! They are given a room in this serai, and perhaps a whole family, including camels and donkeys, will live there while they [the hospital] attend to the patient, sometimes for a month at a time! The hospital feeds the patient, but the family makes its own cooking arrangements. The patients are from Yakam, or Tibet, or Persian-speaking Afghans from beyond Kabul, and, of course, often Mohmunds and Mushuds and all the Khels (tribes) of the border." This glimpse of the C.M.S. Peshawar Hospital is only another instance of how much the Missionary Societies have lost in the past by neglecting to publish first-hand and detailed accounts of the conditions of hospital work on the edges of civilisation. Since the above was written the Church Missionary Society has received news that on March 17 a member of the staff of the Peshawar Hospital was fatally stabbed. The victim was Dr. Vernon Harold Starr, who joined the staff of the institution in 1910. His fate is peculiarly sad, since his influence on the population was highly valued. The only explanation so far is that he was stabbed by a fanatic.

